# Supplementary material for: Syngeneic N1-S1 Orthotopic Hepatocellular Carcinoma in Sprague Dawley Rat for the Development of Interventional Oncology-Based Immunotherapy: Survival Assay and Tumor Immune Microenvironment
Source: Cancers (Basel). 2023 Jan 31;15(3):913. doi: 10.3390/cancers15030913 (PMC9913283; doi:10.3390/cancers15030913)
Supplement: Supplementary file 1 [file cancers-15-00913-s001.zip › cancers-2114395-supplementary.pdf]

Supplementary Information for

Syngeneic N1-S1 Orthotopic Hepatocellular Carcinoma in Sprague Dawley Rat for the Development of Interventional Oncology-based Immunotherapy: Survival assay and Tumor Immune Microenvironment

Bongseo Choi[1+], Jason Pe[1+], Bo Yu[1], and Dong-Hyun Kim,[1,2,3,4*]*

[1]Department of Radiology, Feinberg School of Medicine, Northwestern University, Chicago, IL 60611, USA
[2]Department of Biomedical Engineering, McCormick School of Engineering, Northwestern University, Evanston, IL 60208, USA
[3]Robert H. Lurie Comprehensive Cancer Center, Northwestern University, Chicago, IL 60611, USA
[4]Department of Biomedical Engineering, University of Illinois at Chicago, Chicago, IL 60607, USA

+ These authors contributed equally to this work.
***Corresponding Authors:** Dr. Bo Yu (bo.yu@northwestern.edu) and Prof. Dong-Hyun Kim (dhkim@northwestern.edu)

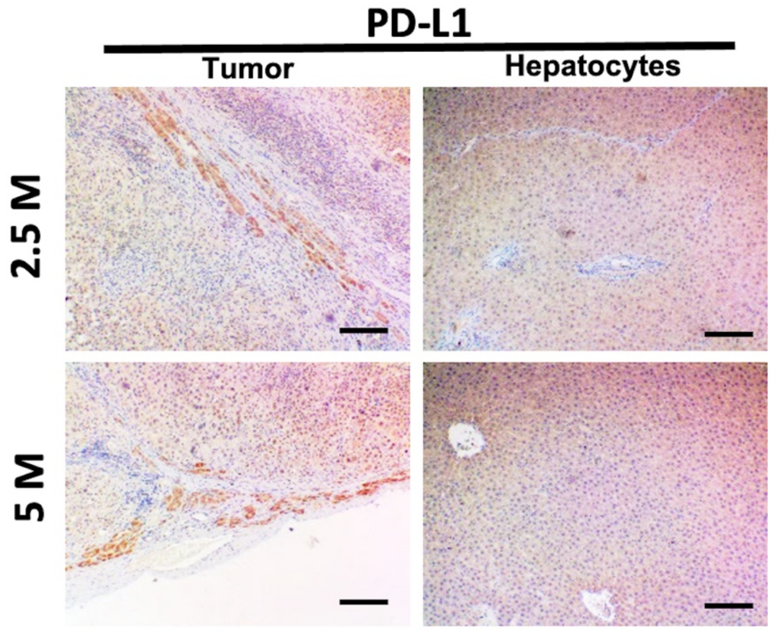

Figure S1. Immunohistology analysis of N1-S1 HCC tumor generated with a subcapsular N1-S1 cells (2.5 and 5 million N1-S1 cells) implantation in Sprague Dawley rats. PD-L1 (positive brown signal) stained images of tumor and peripheral hepatic region. Scale bars: 150 μm.

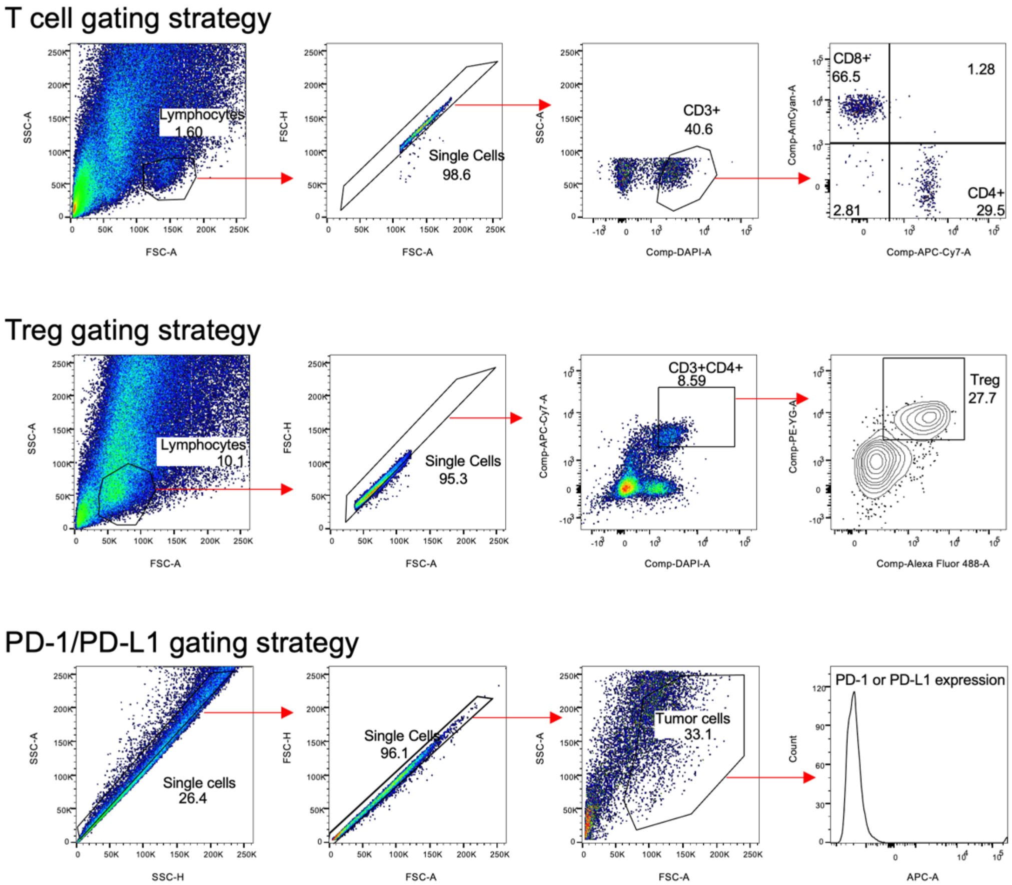

Figure S2. Gating strategies for flowcytometry analysis. All samples were primarily gated from the ly mphocytes and single cells gates.

Table S1. Antibodies used for flowcytometry

Antibody	Provider	Clone	Catalogue Number	Dilution	Identification of what cell phenotypes
CD3-PE	Biolegend	1F4	201412	1:400 (0.5 ug/mL)	T lymphocyte
CD4-PEcy7	Biolegend	W3/25	201516	1:200 (1 ug/mL)	Peripheral T cell subset (helper T cells)
CD25-APCcy7	Biolegend	OX-39	202114	1:200 (1 ug/mL)	IL-2 receptor α chain
FoxP3-PECF594	Biorbyt	Polyclonal	orb156940-CF594	1:500 (1 ug/mL)	Forkhead box P3 (regulatory T cells)
PD-1-APC	Bioss	Polyclonal	Bs-1867R-A647	1:200 (5 ug/mL)	PD-1/CD279
PD-L1-APC	NovusBio	Polyclonal (6H12)	NBP1-76769APC	1:100 (2.5 ug/mL)	PD-L1/CD274
CD8-FITC	LSBio	Monoclonal	LS-C12709-50	1:10 (10 ug/mL)	Peripheral T cell subset (cytotoxic T cells)
